# A novel non-surgical method for mild peri-implantitis- a multicenter consecutive case series

**DOI:** 10.1186/s40729-017-0098-y

**Published:** 2017-08-03

**Authors:** J. C. Wohlfahrt, B. J. Evensen, B. Zeza, H. Jansson, A. Pilloni, A. M. Roos-Jansåker, G. L. Di Tanna, A. M. Aass, M. Klepp, O. C. Koldsland

**Affiliations:** 1 Department of Periodontology, Institute of Clinical Dentistry, University of Oslo, Pb. 1109 Blindern, 0317 Oslo, Norway; 2 Private Practice, Tønsberg, Norway; 3 Department of Dental and Maxillofacial Sciences, Section of Periodontology, Sapienza, University of Rome, Rome, Italy; 4 Center for Oral Health, Department of Natural Science and Biomedicine, School of Health Sciences, Jönköping University, Jönköping, Sweden; 5 Department of Periodontology, Public Dental Health Service, Kristianstad, Sweden; 6 Center for Primary Care and Public Health, Queen Mary University of London, London, UK; 7 Private Practice, Stavanger, Norway; 8 Department of Periodontology, Public Specialist Dental Clinic, Karlskrona, Sweden

**Keywords:** Clinical study, Chitosan, Peri-implantitis, Dental implants, Non-surgical treatment

## Abstract

**Aim:**

The aim of the present study was to evaluate the effect on peri-implant mucosal inflammation from the use of a novel instrument made of chitosan in the non-surgical treatment of mild peri-implantitis across several clinical centers.

**Materials and methods:**

In this 6-month multicenter prospective consecutive case series performed in six different periodontal specialist clinics, 63 implants in 63 patients were finally included. The subjects had mild peri-implantitis defined as radiographic bone loss of 1–2 mm, pocket probing depth (PPD) ≥4 mm and a positive bleeding on probing (mBoP) score. The patients were clinically examined at baseline and after 2, 4, 12 and 24 weeks, and radiographs were taken at baseline and at 3 and 6 months. Treatment of the implants with the chitosan brush seated in an oscillating dental drill piece was performed at baseline and at 3 months. Reductions in the clinical parameters (PPD and mBoP) were compared between baseline and the later examination time points.

**Results:**

Significant reductions in both PPD and mBoP were observed at all time points compared with the baseline clinical measurements (*p* < 0.001). The mean PPD and mBoP at baseline were 5.15 mm (4.97; 5.32) and 1.86 (1.78; 1.93), respectively, whereas the mean PPD and mBoP at 6 months were 4.0 mm (3.91; 4.19) and 0.64 (0.54; 0.75), respectively. Stable reductions in PPD and mBoP were evident up to 6 months after the initial treatment and 3 months after the second treatment. All 63 implants were reported to have stable radiographic levels of osseous support.

**Conclusions:**

This case series demonstrated that an oscillating chitosan brush is safe to use and seems to have merits in the non-surgical treatment of dental implants with mild peri-implantitis. To measure the effectiveness of the method, a multicenter randomized clinical trial needs to be undertaken.

## Background

Inflammation and loss of attachment around dental implants (i.e. peri-implantitis) has become a growing concern within the field of dental implantology [1–7]. Peri-implantitis is a microbial infection-driven soft tissue inflammation with loss of bony attachment around an implant. Peri-implant mucositis is the precursor of peri-implantitis, as gingivitis is for periodontitis [8]. It is clearly shown that daily infection control performed by the patient and regular professional maintenance of dental implants is important to prevent the progression of mucositis to peri-implantitis [9–12]. In advanced cases, peri-implantitis may lead to implant loss.

The current view is that most cases of peri-implantitis are unmanageable without surgical intervention. However, the stage of disease progression at which surgery is necessary remains undefined, and limited scientific evidence is available regarding surgical methods that hinder the progression of the disease over time. Because peri-implantitis surgery often involves a high level of patient morbidity, the development of non-surgical and less-invasive treatment methods is of interest for both patients and the dental community. Currently used methods for non-surgical implant debridement include titanium curettes, plastic or carbon fibre curettes, ultrasound, air-polishing and lasers. No particular non-surgical treatment for peri-implantitis resulting in superior outcomes is however supported by sufficient scientific evidence [13, 14]. Furthermore, some procedures have been suggested to cause more problems rather than improving peri-implant health [15]. The crux is to intervene and to treat the inflammation without causing further problems that may contribute to the progression of peri-implant attachment loss. For example, leaving remnants of an instrument could cause a foreign body reaction, which may accelerate disease progression and attachment loss [16]. Similarly, using an ultrasonic steel tip may induce damage to the titanium surface whereas a nylon tip may result in melted material remnants on the implant surface [17]. In a recent study by Eger et al. [15], it was reported that debridement of titanium surfaces with an ultrasonic device may release titanium particles that was shown to induce a pronounced inflammatory response which caused osteoclastogenesis. The use of ultrasonic devices on titanium surfaces may thus aggravate peri-implantitis rather than resolve the situation.

A number of other studies also report that leaving fragments of the instrument on the implant surface or scratching the surface may impede optimal peri-implant healing [17–20].

Chitosan is a marine biopolymer which is based on chitin derived from the shells of marine crustaceans. The material has been approved for use in surgical bandages, as a haemostatic agent and as a dietary supplement in a wide range of nutritional and health products. Chitosan has also been documented to be non-allergenic and may exhibit anti-inflammatory properties. The material is demonstrated to be completely biocompatible which also recently was verified in an animal experimental study [21]. In a pilot randomized split mouth clinical trial including 11 patients with mucositis, it was demonstrated that debridement with a chitosan device or titanium curettes lead to significant reduction in peri-implant mucositis. A better reduction in parameters of inflammation was however seen at 4 weeks at the implants treated with the chitosan device as compared with titanium curettes (Wohlfahrt et al. submitted for publication).

In the present study, the aim was to evaluate the effect on implant mucosal inflammation from the use of a novel instrument made of chitosan in the non-surgical treatment of mild peri-implantitis across several clinical centers.

## Case presentation

### Materials and methods

A 6-month multicenter prospective consecutive case series was performed in six different periodontal specialist clinics in Norway, Sweden and Italy.

Ethical approval was provided by the regional ethical review boards of each center (Norway: 2014/852/REK sør-øst; Italy: Sapienza 2011/15, 3547; and Sweden: EPN Lund 2014/695.) Fifteen patients at each center were planned to be included in the study. Patient screening, inclusions and all clinical examinations were performed by board-certified specialists in periodontology. Subjects were included in the study if they had at least one implant that had been in function for more than 12 months and had been diagnosed with mild peri-implantitis defined as 1–2-mm bone loss, pocket probing depth (PPD) ≥4 mm and a positive bleeding on probing score. Patients diagnosed with periodontitis were required to be finished with active periodontal treatment prior to inclusion in the study. All six surfaces of the included implants were free of supragingival visible plaque. Patients were required to have a total plaque score (dichotomous scoring) below 20% prior to inclusion, and baseline measurements were performed after careful oral hygiene instruction on an individual, as-needed basis. Radiographs were taken at baseline and at the 6 months evaluation. Endodontic lesions and dental decay should have been treated prior to inclusion. Clinical examinations were performed at baseline and at 2, 4, 12 and 24 weeks after baseline using a 0.20-N (20-g)-defined force periodontal probe (University of North Carolina, DB764R, AESCULAP, B Braun Germany). PPD and mBoP was recorded at six sites per implant. Bleeding on probing (mBoP) was recorded using a 3-graded index 30 s after probing as follows: A score of 0 represented no bleeding, 1 represented isolated minimal bleeding spots, 2 represented blood forming a confluent red line on the margin and 3 represented heavy or profuse bleeding [22]. The clinical protocol also included scoring of the height of the gingival margin relative to the crown margin.

All patient-related information and clinical recordings were recorded in a web-based clinical research form (VieDoc version 3.24, PCG solutions, Uppsala, Sweden).

Patients under 18 years of age; current smokers; patients who had undergone radiotherapy in the head and neck region, chemotherapy or systemic long-term corticosteroid treatment; patients who were pregnant or nursing; patients receiving medications known to induce gingival hyperplasia; patients with uncontrolled diabetes (HbA1c >6.5); patients who had taken systemic antibiotics less than 6 months prior to baseline; patients receiving bisphosphonate treatment; and patients with prosthetic factors that prevented clinical measurements were excluded from participation in the study. Implants with technical complications, such as loose supraconstructions, cement remnants, ill-fitted crowns with poor marginal contour or any type of prosthetic complication that according to the examiner, would be a local contributing factor to inflammation, were also excluded. Implants that were previously treated surgically for peri-implantitis and implants with overcontoured supraconstructions obstructing access for debridement and clinical measurements of more than three surfaces were also excluded. Before agreeing to participate in this study, all patients were provided with sufficient information via a patient information sheet and a consent form, which was signed prior to final inclusion.

**Fig. 1:**
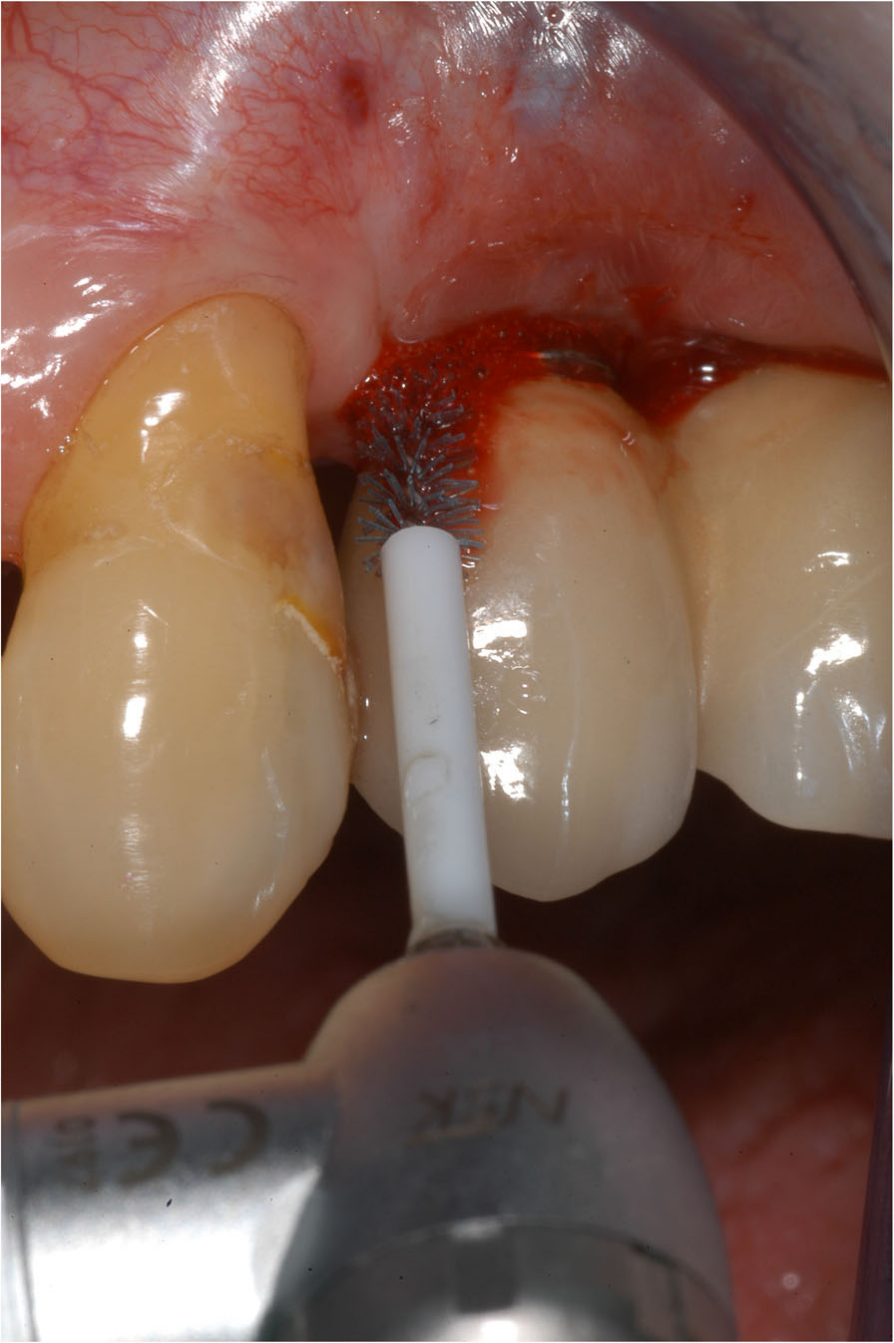
A chitosan brush (LBC, BioClean®, LABRIDA AS) seated in an oscillating dental handpiece

After clinical recordings, the implant pockets were debrided with a chitosan brush (LBC, BioClean®, LABRIDA AS, Oslo, Norway) seated in an oscillating dental drill piece (ER10M, TEQ-Y, NSK Inc., Kanuma Tochigi, Japan) for 3 min and then irrigated with sterile saline (Fig. 1). The initial debridement was performed with local anaesthesia as needed. A second debridement with the chitosan brush was performed after 3 months.

### Statistical analysis

The power calculation was based on data from a pilot clinical study evaluating the same test device performed at the Department of Periodontology, University of Oslo, in 2014. Descriptive statistics were presented for continuous variables (median and interquartile range), and proportions were presented for categorical variables.

Mann-Whitney rank sum tests were used to compare changes in the clinical parameters between baseline and subsequent time points. To assess the hierarchical structure of the data (center > patient > site), a linear mixed model using the restricted maximum likelihood method (multilevel logistic models for binary outcomes) was constructed to analyse the PPD, mBoP and suppuration, adjusting for factors such as age, gender and past smoke exposure.

All statistical analyses were performed using Sigma Plot v 13.0 (Systat Software, Inc., San Jose, CA) and Stata 14 (StataCorp, College Station, TX, USA) statistical software.

## Results

In total, 63 implants in 63 patients were ultimately included in the analysis. Demographic information is presented in Tables 1 and 2.

**Table 1:** Demographics

Variable	Number (%)	SD	Range (min; max)
Gender (female/male)	45/18 (71.4/28.6)		
Age	58.4	14.4	23; 85
Former smokers	39 (62.1)		
Implant age	8.9	6.9	1.5; 30
Implant brand			
ASTRA	12 (19.0)		
NOBEL	27 (42.9)		
Straumann	7 (11.1)		
Sweden and Martina	2 (3.2)		
TMI	2 (3.2)		
Implandent	1 (1.6)		
Friadent	1 (1.6)		
Unknown	11 (17.5)

**Table 2:** Demographics by center

Center	Oslo	Jonkoping	Rome	Stavanger	Kristianstad	Tonsberg	Total
Number of patients	12	11	12	3	11	14	63
Gender (f)	9	5	11	2	6	12	45
Age	60(26–85)	60(23–73)	55(29–77)	49(41–55)	61(36–73)	57(26–82)	63(26–85)
Implant age	12(2.3–30)	6.2(1.5–21.3)	10(2–21)	11(7.9–17)	8(1.5–23)	5.5(2.3–10.3)	8.7(1.5–30)
Tooth loss							
Agenesis	2	1	1	1	1	0	6
Caries	2	3	3	0	0	1	9
Endodontics	0	0	0	0	0	2	2
Periodontitis	4	0	4	1	3	1	13
Trauma	2	2	3	1	0	0	8
Other	2	5	1	0	7	10	25

**Fig. 2:**
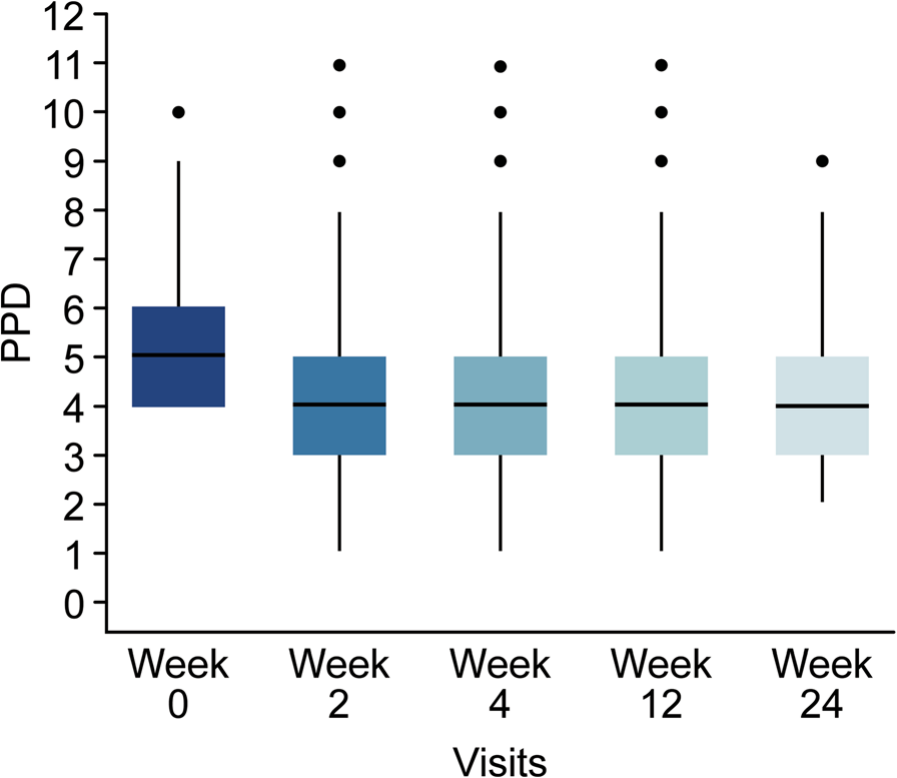
Changes in PPD values between baseline and the various examination time points

**Fig. 3:**
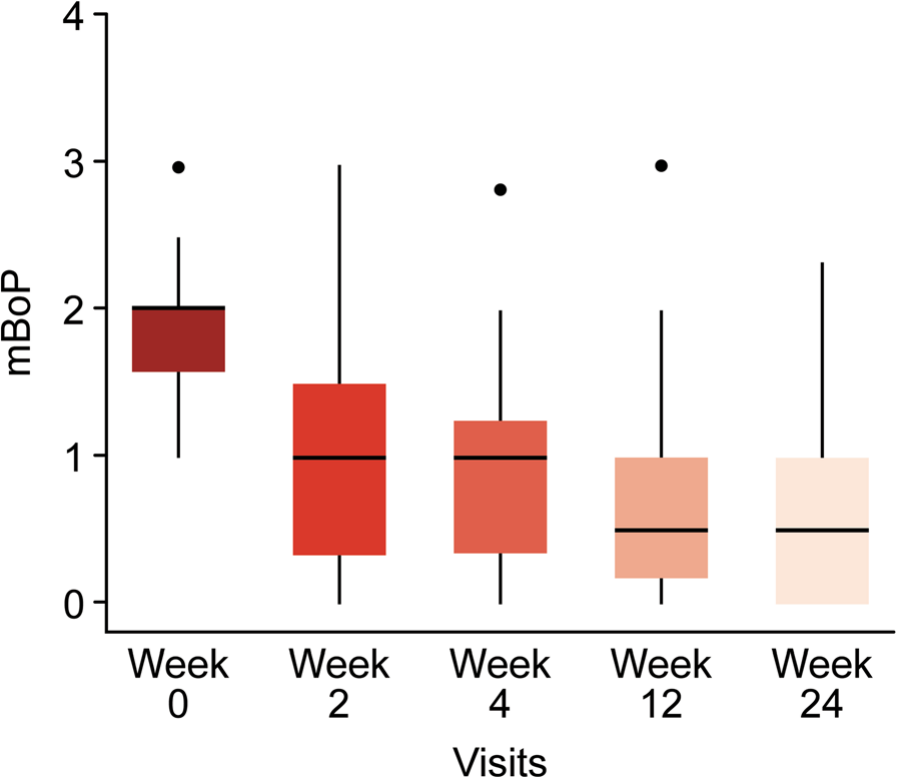
Changes in BoP values between baseline and the various examination time points

Significant reductions in both PPD and mBoP were seen at all time points relative to the baseline clinical measurements (*p* < 0.001) in both the unadjusted and adjusted models including age, gender and past smoking experience (Figs. 2 and 3). Stable reductions in PPD and mBoP were observed after 2 weeks and up to 6 months after the initial treatment. The hypothesis of no difference in PPD values between week 2 and week 4 (*p* = 0.1429) could not be rejected, but from week 4 until 3 and 6 months, statistically significant reductions with *p* values of 0.007 and 0.0295, respectively, were detected. A relevant cluster effect at the center level was identified with variation around the center intercepts of 0.267 and 0.117 and remaining variances of 0.432 and 0.46 for PPD and mBoP, respectively.

The mean PPD and mBoP at baseline were 5.15 mm (4.97; 5.32) and 1.86 (1.78; 1.93), respectively, whereas the mean PPD and mBoP at 6 months were 4.0 mm (3.91; 4.19) and 0.64 (0.54; 0.75), respectively (Figs. 2 and 3).

**Fig. 4:**
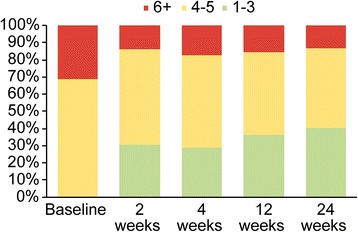
Percentages of sites with PPD 1–3, 4–5 and ≥6 mm by visit (*p* < 0.0001)

A mBoP index of 1 or more and PPD ≥4 mm was recorded at 35% of the sites at the final examination. At the baseline examination, PPD ≥6 mm was recorded at 31.25% (25.53; 37.59) of the sites and 17.02% (8.14; 31.35) of the implants. These numbers were reduced to 13.25% (9.31; 18.43) of the sites and 4.35% (0.76; 16.04) of the implants at the terminal examination (Fig. 4).

**Fig. 5:**
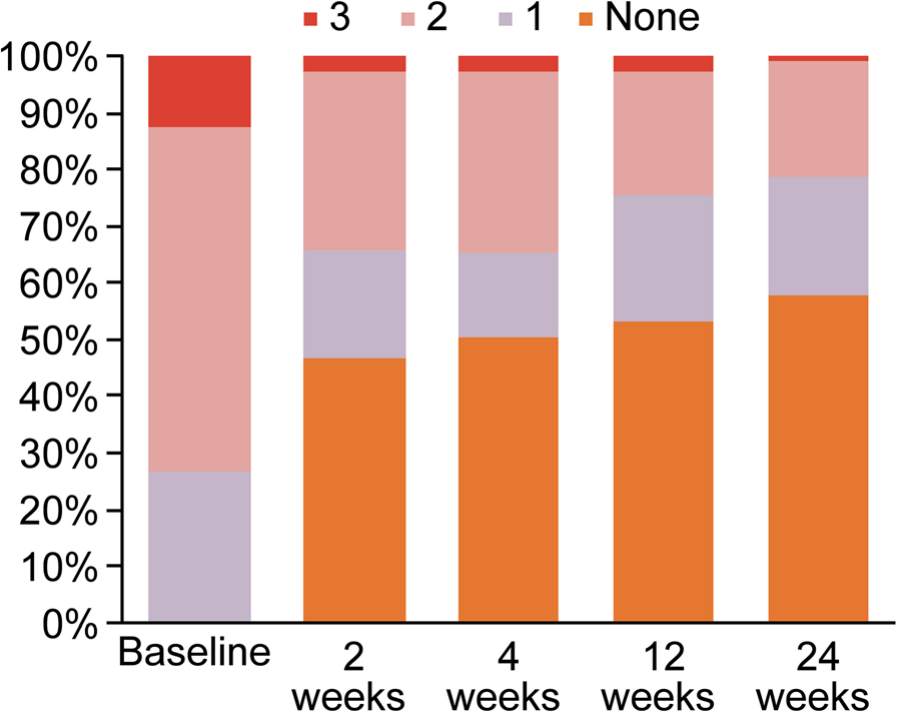
Change in BoP values according to the percentage of sites with a score of 1, 2 or 3 by visit

At the baseline visit, a mBoP index of 2 or 3 was recorded at 73.14% (67.01; 78.52) of the included sites, while this number was reduced to 28.9% (16.3; 27.1) at the final evaluation (Fig. 5).

Statistical difference in the level of the mucosal margin was recorded between baseline and all the later time points. No further change was seen after 4 weeks (Table 3).

**Table 3:** Level of crown margin at the different time points

	Baseline *n* = 306	2 weeks *n* = 272	4 weeks *n* = 267	12 weeks *n* = 282	24 weeks *n* = 294	*P*
Subgingival crown margins	283 (92.5%)	224 (82.4%)	224 (83.9%)	239 (84.8%)	248 (84,4%)	
Supragingival crown margins	23 (7.5%)	48(17.6%)	43 (16.1%)	43 (15.2%)	46 (15.6%)	
Baseline to 2 weeks						<0.001
Baseline to 4 weeks						0.024
Baseline to 12 weeks						0.003
Baseline to 24 weeks						0.002
4 to 12 weeks						0.668
12 to 24 weeks						0.895

During this study, all 63 implants were reported to have stable radiographic levels of osseous support as validated by the six different local examiners. No adverse events were reported during the study.

## Discussion

Identifying peri-implant disease at an early stage and promptly treating the inflammatory condition is crucial to prevent the progression of peri-implant bone loss and ensure long-term implant survival [23–25]. After completion of active treatment and when the condition is controlled, supportive peri-implant therapy will reduce the risk of disease re-occurrence [9]. A number of scientific reports on various methods for non-surgical peri-implant therapy have been presented, but limited and short-term effects have been reported [26, 27]. Instruments for the removal of submucosal microbial deposits from implant surfaces should obviously be effective without causing damage to the implant. However, clinical devices specifically designed for this purpose are scarce, and the effectiveness and safety of most such devices have rarely been scientifically validated [28]. In a review paper by Schwarz et al. [29], it was reported that mechanical debridement with, e.g. carbon fibre, titanium or plastic curettes combined with measures of oral hygiene, was effective in the management of peri-implant mucositis and that alternative or adjunctive measures such as lasers, ultrasonic devices or air abrasives with glycine powder may improve the efficacy of the treatment of sites with peri-implantitis. The same group of researchers also performed a systematic review on studies evaluating air-polishing with glycine powder of implants with peri-implantitis and reported that this method may lead to improved reduction in parameters of inflammation as compared to mechanical debridement combined with antiseptic therapy [30]. Chitosan is a completely biocompatible biopolymer which also has been demonstrated to be bacteriostatic and exhibit anti-inflammatory properties [31–33]. A recent in vitro experimental study demonstrated that chitosan inhibits the growth of the periodontal pathogens *Porphyromonas gingivalis* and *Aggregatibacter actinomycetemcomitans* and exerts an anti-inflammatory effect by reducing the levels of prostaglandin E-2 (PGE_2_) [34]. From these perspectives, chitosan may be considered a potential candidate to be used in a device for implant debridement.

In the present study, significant reductions were observed in the clinical parameters of peri-implant inflammation at 2, 4, 12 and 24 weeks relative to baseline after debridement with the chitosan brush seated in an oscillating dental drill piece. No progression in radiographic bone loss was reported at any of the implants at the final evaluation, and the method was thus judged safe to use in cases with mild peri-implantitis.

In comparison, a randomized clinical trial by Sahm et al. [35] compared amino acid glycine powder versus mechanical debridement using carbon curettes and antiseptic therapy with chlorhexidine digluconate. At the 6-month final evaluation, PPD reductions of 0.6 and 0.5 mm, respectively, were reported. Similarly, Renvert et al. [36] performed a randomized clinical trial comparing an air-abrasive device and an Er:yttrium aluminium garnet (YAG) laser in the non-surgical treatment of peri-implantitis and reported mean PPD reductions of 0.9 and 0.8 mm, respectively, in the two groups. In the present study, a mean PPD reduction of 1.1 mm was determined at the final evaluation at 6 months which is comparable to findings reported in other studies.

A study by Riben-Grundstrom and co-workers [37] compared the use of glycine powder air-polishing and the ultrasonic treatment of peri-implant mucositis and utilized inclusion criteria comparable to those used in the present study for mild peri-implantitis. The inclusion criteria were (1) the presence of one or more sites diagnosed with peri-implant mucositis. The diagnostic criteria used were a probing depth ≥4 mm combined with bleeding with or without suppuration and (2) bone loss ≤2 mm assessed from the implant shoulder subsequent to the bone healing and remodelling process. They used dichotomous values for BoP and observed reductions of 27% in the air-polishing group and 31% in the ultrasonic group after 6 months.

When pooling the index scores to dichotomous values in the present study, the mBoP score was found to have decreased from 2 or 3 to 0 or 1 in 55% of the samples. Although the complete absence of inflammation was difficult to achieve in most implants, significant and stable reductions in the parameters of inflammation were demonstrated in most sites up to 6 months after treatment with the chitosan brush. Nicotine interferes with the bleeding response in soft tissues and may, consequently, lead to false negatives for the BoP; therefore, to avoid positively skewing the results due to smoking, we decided to exclude current smokers from this study. According to the literature, bleeding on probing has low sensitivity as a predictor for active peri-implant disease because of the high frequency of false-positive responses, but it has a high level of specificity as no bleeding on probing indicates peri-implant health [38]. Due to the absence of perpendicular periodontal fibres in dental implants, a lighter probing force should be used than when probing the gingival crevice in teeth. Similarly, the standardization of the examiners’ technique is critical [39]. A pressure-sensitive periodontal probe was used to record PPD and mBoP. We also used a 3-graded bleeding index to further distinguish between true disease and bleeding from the base of the pocket as the result of excessive pressure and rupture of the junctional epithelium. Scores of 1 and 0 and scores of 2 and 3 were pooled to create a more rigid, dichotomous score. This strengthens the positive results because significant differences were obtained when both the graded and dichotomous BoP scores were analysed. However, previous smokers were included but the outcomes in this patient group did not differ from finding in never smokers. Similarly, patients taking anticoagulants were excluded to avoid false-positive bleeding scores because of the increased bleeding response caused by the medication. Salvi and co-workers [40] studied the reduction in experimental peri-implant mucositis and revealed that 3 weeks of reinstituted plaque control did not yield pre-experimental levels of peri-implant health. While infection control was carefully installed prior to baseline, and the included implants were plaque free, we found significant reductions in the parameters of inflammation as early as 2 weeks after treatment with the chitosan brush. These results were stable up to 6 months after treatment, indicating a fast and stable response. More of the crown margins were exposed 2 weeks after debridement with the brush and the levels were thereafter stable. The more apical position the crown margins is most probably related to reduction in the soft tissue oedema from the inflammation.

The chitosan brush used in this study is made of a material that is soft with the aim to make a device optimized for removal of the biofilm within the implant threads. The soft bristles on the contrary make the device suboptimal for removal of hard deposits, such as calculus and cement remnants. It has been reported that such cement remnants are a common finding around dental implants [41], and in hindsight, it would have been beneficial for the analysis to record this. We did not record or analyse on cement- or screw-retained supraconstructions, and it may well be that some of the implants with cement-retained crowns and bridges had subgingival and non-visible cement remnants contributing to the mucosal inflammation. It can thus be hypothesized that combining the brush with an instrument for the removal of potential hard deposits would have yielded even better results. One such instrument for surgical use on titanium surfaces is a rotating titanium brush. The disadvantage with such a rigid metal brush is that the metal bristles may potentially cause injure to the mucosa if used non-surgically. Once active peri-implantitis treatment is finished with a positive response verified and the long-term and regular supportive treatment phase initiated, it could be argued that avoiding instruments that may damage the implant surface is preferable [15]. The consecutive case series presented here does only show the potential merits of a chitosan brush on reducing peri-implant mucosal inflammation. No control group was included in this study, and to test the clinical efficacy of this novel device in the non-surgical treatment of peri-implantitis, a randomized clinical trial will be required. It is also important with a long-term follow-up to study if the use of this novel device will prevent progression of peri-implant bone loss over time, potentially causing implant loss.

## Conclusions

In this multicenter case series of implants affected by mild peri-implantitis, significant reductions in the clinical parameters of inflammation were demonstrated at all time points after the initial treatment with a chitosan brush. The use of an oscillating chitosan device appears to be safe and has potential merits for the treatment of mild peri-implantitis and for the maintenance of dental implants. To measure the effectiveness of the method, a multicenter randomized clinical trial needs to be undertaken.
